# Epidemiological trends of reported syphilis incidence in Xihu district, Hangzhou, China, 2005–2025

**DOI:** 10.3389/fepid.2026.1944790

**Published:** 2026-09-11

**Authors:** Jing Li, Luyang He, Tingting Wu, Jiajia Meng, Taoran Wang, Fei Sun, Bangma Wang

**Affiliations:** Hangzhou Xihu District Center for Disease Control and Prevention (Hangzhou Xihu District Health Supervision Institute), Hangzhou, China

**Keywords:** age disparity, epidemiological trend, gender difference, Hangzhou, incidence, joinpoint regression, syphilis

## Abstract

**Background:**

Syphilis remains a critical global public health concern. The reported incidence of syphilis in Zhejiang Province has long stayed at a high level nationwide, with Xihu District of Hangzhou identified as a hyperendemic area for local syphilis transmission. This study used 2005–2025 surveillance data of Xihu District, Hangzhou to analyze syphilis trends for precise prevention evidence.

**Methods:**

Syphilis surveillance data (2005–2025) for Xihu District were retrieved from China's National Notifiable Infectious Disease Reporting System. We calculated crude reported incidence rate for overall, primary, secondary, tertiary, and latent syphilis (per 100,000 population), and notification rate for congenital syphilis (per 100,000 live births). Joinpoint Regression 6.0.1 identified trend inflection points and computed period-specific annual percentage change (APC) and overall average annual percentage change (AAPC) across the 21-year span.

**Results:**

Total syphilis reported incidence rate presented an inverted U-shaped trajectory: rising rapidly from 68.71/100,000 in 2005 to the peak of 155.16/100,000 in 2010, followed by sustained sharp decline down to 13.17/100,000 in 2025, with the overall AAPC = −8.18% (*p* < 0.05). Four trends phases were identified: rapid growth (2005–2008, APC = 25.23%), mild decline (2008–2019, APC = −5.00%), accelerated decline (2019–2023, APC = −18.69%), and apparent reduction (2023–2025, APC = −39.01%). Latent syphilis dominated all stages throughout the study period. Primary and secondary syphilis shared long-term descending trends (AAPC = −14.76% and −12.19%, respectively). Tertiary syphilis remained at extremely low absolute levels with sparse case counts. Congenital syphilis surged in 2006–2007 and no notified congenital syphilis cases have been recorded since 2017. Gender disparity existed: females exhibited a faster overall descending rate (AAPC = −10.23%) than males (AAPC = −5.54%). The 20–39 age group consistently bore the highest reported incidence burden, with a long-term AAPC of −10.47%; the elderly (≥60 years) also showed prominent high reported incidence before 2019 and then dropped sharply (AAPC = −9.53%).

**Conclusions:**

Syphilis control achieved substantial progress in Xihu District over two decades. Still, epidemiological challenges remain, including persistent vulnerability among young and older adults and hidden undiagnosed latent infections. Tailored interventions for high-risk groups, standardised latent-syphilis care, and sustained prevention of mother-to-child transmission are needed to lower residual disease burden.

## Background

Syphilis, caused by the spirochete Treponema pallidum, remains a major public health challenge worldwide despite being readily treatable with penicillin. The global burden of syphilis has shown concerning trends, with an estimated 7.1 million new cases among adults aged 15–49 years in 2020, representing an increase from previous estimates (1). The World Health Organization (WHO), in its Global Health Sector Strategies on HIV, Viral Hepatitis and Sexually Transmitted Infections for the period 2022–2030, has set ambitious targets for 2030, namely a 90% reduction in adult syphilis incidence relative to the 2020 baseline, a congenital syphilis case rate of fewer than 50 per 100,000 live births (2).

China has experienced a significant syphilis resurgence since the late 1990s, driven by rapid socioeconomic transformation, urbanization, changing sexual norms, and the expansion of mobile populations (3). A national study examining notifiable sexually transmitted infections between 2006 and 2022 found that syphilis had the highest average yearly incidence among all Sexually Transmitted Infections (STIs) in China, at 28.75 cases per 100,000 population, with increasing trends observed among adolescents, the elderly, and males (4). The epidemiology of syphilis, however, shows substantial geographical heterogeneity across China's diverse regions, necessitating local-level analyses to inform context-specific prevention strategies.

The reported incidence of syphilis in Zhejiang Province, China, has remained relatively high nationwide for a long time (5), and comprehensive syphilis prevention and control has always served as a core task of public health governance in the province. Previous studies have verified that Xihu District of Hangzhou is a high-risk region for syphilis transmission in Zhejiang Province (6). As the provincial capital of Zhejiang Province and one of the most economically dynamic cities in China, Hangzhou has experienced accelerated urbanization and continuous population expansion over the past two decades. Located in the central urban area of Hangzhou, Xihu District is characterized by dense resident population, massive inflow of migrant population and a sound healthcare system, which creates a distinctive epidemiological setting. Systematic analysis of the long-term epidemiological characteristics of syphilis in this district can scientifically evaluate the effectiveness of existing prevention and control measures, accurately identify deficiencies and gaps in current intervention practices, and thus provide valuable references for public health practice.

Previous studies have respectively explored the temporal trends and spatiotemporal distribution patterns of syphilis incidence in Zhejiang Province during 2016–2022 (7) and 2005–2018 (6) separately. Nevertheless, long-term epidemiological analyses covering more than two decades remain scarce, and in particular, few research has addressed the long-term epidemiological trends of syphilis stratified by gender and age groups across Zhejiang Province. Given the dynamically evolving epidemiological landscape of syphilis and the pivotal role of region-specific baseline data in evidence-based public health decision-making, this study adopts 21 consecutive years of syphilis surveillance data spanning 2005 to 2025 from Xihu District, Hangzhou. It systematically characterizes the temporal variation of the reported syphilis incidence rate in this district, further analyzes epidemiological features stratified by clinical stage, gender and age cohorts, identifies high-risk priority subpopulations and critical time windows for targeted precision interventions, and thereby provides empirical evidence for the formulation of differentiated prevention and control strategies.

## Methods

### Study setting and data sources

Data on all reported syphilis cases in Xihu District between January 1, 2005, and December 31, 2025, were obtained from the China Information System for Disease Control and Prevention (CISDCP). This passive surveillance system was formally launched after the 2003 SARS epidemic and provides a real-time online reporting platform for all notifiable infectious diseases (8). Hospitals, community health centers, and township health facilities are required to report confirmed syphilis cases within 24 h of diagnosis, based on standardized clinical and laboratory diagnostic criteria. Data on all notified syphilis cases from 2005 to 2024 were complete full-year records. The 2025 surveillance dataset was fully collected, audited and finalized by December 31, 2025 without delayed case entry or underreporting bias. “All syphilis” in the present study aggregates primary, secondary, tertiary, and latent syphilis; congenital syphilis is excluded from this composite measure owing to its distinct denominator (live births instead of general resident population).

Prior to 2007, all reported cases of syphilis were diagnosed and classified by subtype in accordance with the Chinese National Diagnostic Criteria and management of Syphilis (GB 15974-1995) (9); From 2007 to 2017, diagnosis and subtyping were conducted in accordance with the National Diagnostic Criteria for Syphilis (WS 273-2007) (10); since 2018, diagnosis and subtyping have been conducted in accordance with the National Diagnostic Criteria for Syphilis (WS 273-2018) (11).

### Statistical analysis

Population denominators for reported incidence calculations were annual mid-year stratified population estimates by age and sex, obtained from Hangzhou Municipal Bureau of Statistics. All non-congenital syphilis reported incidence rates (per 100,000 population) were computed as (annual new case count/annual mid-year resident population) × 100,000; congenital syphilis rates were separately calculated per 100,000 live births from district maternal-child health records. The resident population covers all persons living in Xihu District for ≥6 months, including both local registered residents and migrant populations. All syphilis cases were grouped according to patients' current residential address at diagnosis, matching the statistical standard of the resident population denominator. Age-stratified rates were further generated for four subgroups: <20, 20–39, 40–59, and ≥60 years. Temporal trends in reported incidence rates were analyzed using joinpoint regression analysis (Joinpoint Regression Program, Version 6.0.1, National Cancer Institute). This method identifies statistically significant changes in linear trends over time and calculates the annual percentage change (APC) for each trend segment, as well as the average annual percentage change (AAPC) for the entire study period (12).

A log-linear regression model was fitted to temporal reported incidence trends, as presented in Equation 1.

$$\begin{array}{c} \ln\;(\mathrm{rate})=\beta_{0}+\beta_{1}\times \mathrm{Year}+\varepsilon \end{array}$$ (1)

The annual percentage change (APC) for each segmented trend was derived using Equation 2.

$$\begin{array}{c} \mathrm{APC}=(\exp\;(\beta_{1})-\;1)\times 100 \end{array}$$ (2)

where rate is annual syphilis reported incidence rate. Year is time predictor. $\beta_{0}$ is intercept of the log-linear model. $\beta_{1}$ is the regression coefficient corresponding to the year variable (slope). ε is residual error term.

We use the grid search method to identify optimal piecewise connection points. The algorithm systematically evaluated all potential joinpoint locations, calculating the sum of squared errors (SSE) and mean squared error (MSE) for each configuration, with the grid point yielding the smallest MSE selected as the optimal connection point. Following the software developers' recommendations, the model was constrained to a maximum of three joinpoints, with a minimum of two data points required between adjacent joinpoints. The best-fitting model was selected using permutation tests, with statistical significance set at *p* < 0.05. In this study, we noted that our reported incidence data contained some values of zero, and we substituted these zeros with 1% of the smallest incidence. Furthermore, we calculated a false discovery rate-adjusted *p*-value using the Benjamini–Hochberg method (Benjamini et al., 2001) to decrease type-I errors (13). To assess the robustness of our trend findings, we performed two sets of sensitivity analyses. First, we recalculated reported incidence rates using registered household-registration (hukou) population data as an alternative denominator. Second, we adjusted the maximum permitted number of joinpoints (ranging from 1 to 2) to evaluate the stability of joinpoint-regression outputs.

## Results

### Overall temporal trends of syphilis reported incidence stratified by clinical stage, 2005–2025

**Figure 1:**
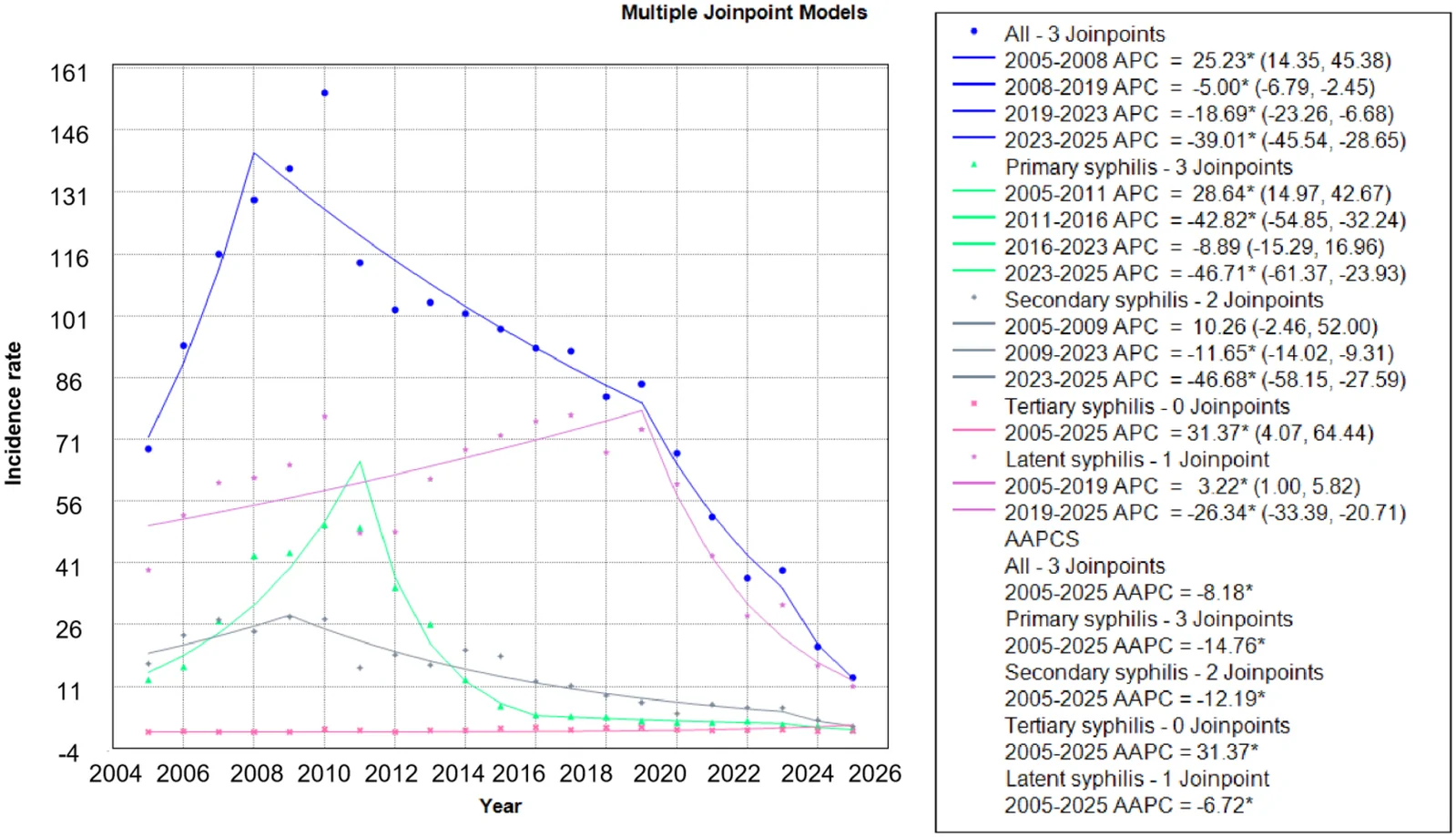
Epidemiological trends of reported syphilis incidence in xihu district, Hangzhou, China, 2005–2025. “All syphilis” comprises primary, secondary, tertiary, and latent syphilis. Reported incidence rates for non-congenital syphilis subtypes are calculated per 100 000 population. The standalone congenital syphilis notification rate is calculated per 100 000 live births. *FDR-adjusted *p* < 0.05.

Table 1 summarises the annual reported incidence rates of all syphilis clinical subtypes in Xihu District, Hangzhou, and Figure 1 visualises their long-term epidemic trajectories. The crude overall syphilis reported incidence followed an inverted U-shaped curve: it climbed steadily from 68.71 per 100,000 in 2005 to a 21-year peak of 155.16 per 100,000 in 2010, before declining continuously to 13.17 per 100,000 by 2025. Joinpoint regression detected three statistically significant inflection points for total syphilis, partitioning the 21-year surveillance period into four distinct trend segments. The reported incidence rose rapidly from 2005 to 2008 (APC = 25.23%, 95% CI: 14.35–45.38, *p* < 0.001), followed by a moderate yet significant decline during 2008–2019 (APC = −5.00%, 95% CI: −6.79 to −2.45, *p* = 0.002). Between 2019 and 2023, this downward trend appeared to accelerate (APC = −18.69%, 95% CI: −23.26 to −6.68, *p* < 0.001); however, the wide confidence interval indicates that the precision of the estimate is limited. The apparent sharp annual decrease across 2023–2025 (APC = −39.01%, 95% CI: −45.54 to −28.65, *p* < 0.001) lacks statistical robustness, as this segment only contains two data points without residual degrees of freedom for variability assessment.

**Table 1:** Reported incidence of syphilis in Xihu district, Hangzhou, China, 2005–2025.

Year	All syphilis		Primary syphilis		Secondary syphilis		Tertiary syphilis		Latent syphilis		Congenital syphilis	
	*N*	IR (per 100,000 population)	*N*	IR (per 100,000 population)	*N*	IR (per 100,000 population)	*N*	IR (per 100,000 population)	*N*	IR (per 100,000 population)	*n*	IR (per 100,000 live births)
2005	417	68.55	77	12.66	101	16.6	0	0.00	239	39.29	1	19.19
2006	557	92.10	96	15.87	142	23.48	1	0.17	318	52.58	10	184.09
2007	678	114.67	159	26.89	161	27.23	0	0.00	358	60.55	8	147.36
2008	772	128.84	256	42.72	146	24.37	0	0.00	370	61.75	2	36.52
2009	795	136.21	254	43.52	163	27.93	0	0.00	378	64.77	3	57.89
2010	910	154.81	295	50.19	161	27.39	4	0.68	450	76.56	2	40.02
2011	869	113.92	378	49.55	119	15.60	3	0.39	369	48.37	0	0.00
2012	782	102.51	268	35.13	143	18.75	0	0.00	371	48.63	0	0.00
2013	801	104.26	201	26.16	125	16.27	3	0.39	472	61.44	0	0.00
2014	785	101.47	98	12.67	154	19.91	3	0.39	530	68.51	1	17.91
2015	757	97.67	49	6.32	143	18.45	7	0.90	558	72.00	1	17.86
2016	736	93.01	33	4.17	97	12.26	9	1.14	597	75.44	1	13.64
2017	745	92.40	31	3.84	90	11.16	4	0.50	620	76.90	0	0.00
2018	686	81.37	31	3.68	74	8.78	9	1.07	572	67.85	0	0.00
2019	730	84.46	23	2.66	62	7.17	10	1.16	635	73.47	0	0.00
2020	615	67.64	21	2.31	41	4.51	5	0.55	548	60.27	0	0.00
2021	568	52.15	25	2.30	73	6.70	4	0.37	466	42.78	0	0.00
2022	413	37.31	30	2.71	66	5.96	5	0.45	312	28.19	0	0.00
2023	437	39.21	21	1.88	66	5.92	6	0.54	344	30.87	0	0.00
2024	234	20.73	17	1.51	33	2.92	2	0.18	182	16.12	0	0.00
2025	155	13.17	6	0.51	16	1.36	3	0.25	130	11.05	0	0.00

*n*, number of cases. IR, reported incidence rate. “All syphilis” comprises primary, secondary, tertiary, and latent syphilis.

Trend patterns differed markedly across clinical syphilis subtypes. The reported incidence rate of primary syphilis grew consistently from 12.66 per 100,000 in 2005 to a peak of 50.19 per 100,000 in 2010, then fell sharply to 0.51 per 100,000 in 2025. Three joinpoints divided its temporal trend into four phases: a significant rising phase from 2005 to 2011 (APC = 28.64%, 95% CI: 14.97–42.67, *p* < 0.001), a steep significant decline spanning 2011–2016 (APC = −42.82%, 95% CI: −54.85 to −32.24, *p* < 0.001), a mild non-significant downward trend between 2016 and 2023 (APC = −8.89%, 95% CI: −15.29 to 16.96, *p* = 0.104), and an apparent precipitous decrease from 2023 to 2025 (APC = −46.71, *p* < 0.001); this two-year segment yields unstable APC estimates with limited interpretability. Among all clinical subtypes, primary syphilis showed the most prominent long-term decline, with an AAPC of −14.76% (*p* < 0.001).

The reported incidence rate of secondary syphilis peaked at 27.93 per 100,000 in 2009 and subsequently declined progressively to 1.36 per 100,000 in 2025. Two joinpoints defined its temporal trajectory: non-significant mild growth during 2005–2009 (APC = 10.26%, 95% CI: −2.46 to 52.00, *p* = 0.089), a prolonged significant downward trend from 2009 to 2023 (APC = −11.65%, 95% CI: −14.02 to −9.31, *p* < 0.001), and an apparent sharp reduction recorded for 2023–2025 (APC = −46.68%, 95% CI: −58.15 to −27.59, *p* < 0.001), yet this terminal two-point segment cannot support reliable long-term trend inference. The full-period AAPC for secondary syphilis stood at −12.19% (*p* < 0.001).

Tertiary syphilis maintained extremely low absolute reported incidence across the whole surveillance period, with five years recording zero reported cases and most years only single-digit case numbers; no statistically meaningful joinpoints were identified. The unified 21-year APC was calculated as 31.37% (95% CI: 4.07–64.44, *p* = 0.021). This wide confidence interval and sparse underlying cases indicate high estimation instability, and this figure cannot be interpreted as a genuine sustained long-term upward epidemic trend, even though the *p*-value reached statistical significance. Latent syphilis remained the predominant clinical subtype every year; its reported incidence increased from 39.29 per 100,000 in 2005 to a subtype-specific peak of 76.90 per 100,000 in 2017, before falling to 11.05 per 100,000 in 2025. A single joinpoint at 2019 split its epidemic curve: slow significant growth from 2005 to 2019 (APC = 3.22%, 95% CI: 1.00–5.82, *p* = 0.018), followed by an apparent rapid significant decline from 2019 to 2025 (APC = −26.34%, 95% CI: −33.39 to −20.71, *p* < 0.001), with an overall AAPC of −6.72% (*p* < 0.001).

Congenital syphilis exhibited a solitary sharp peak of 184.09 per 100,000 in 2006 and declined rapidly each subsequent year; no new congenital syphilis cases were recorded from 2017 through 2025.

### Sex-disaggregated reported incidence and temporal trends of syphilis

**Figure 2:**
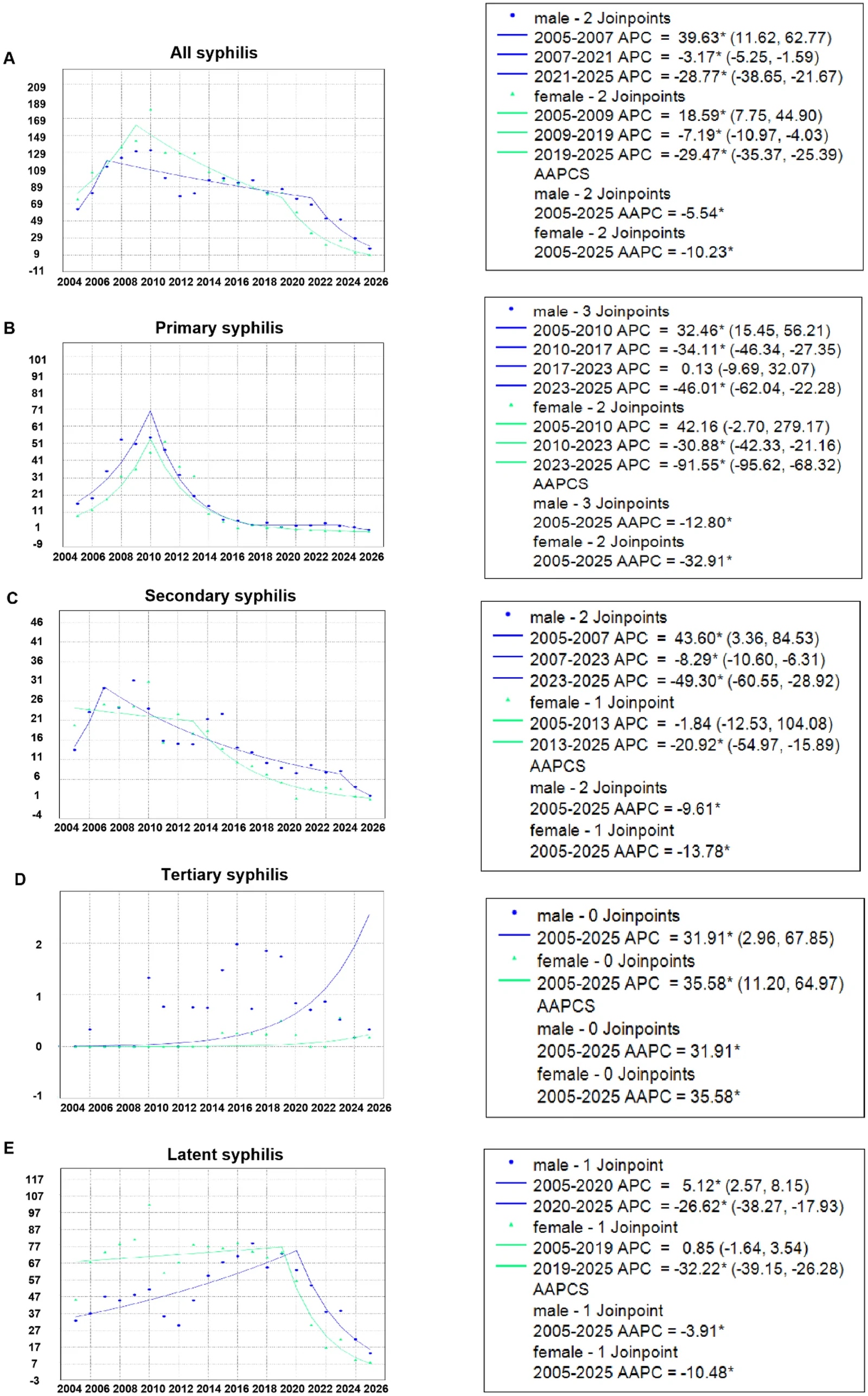
Sex-specific epidemiological trends of reported syphilis incidence in xihu district, Hangzhou, China, 2005–2025. Panels A to E correspond to the incidence rates of all-stage syphilis, primary syphilis, secondary syphilis, tertiary syphilis and latent syphilis, respectively. “All syphilis” comprises primary, secondary, tertiary, and latent syphilis. Reported incidence rates for non-congenital syphilis subtypes are calculated per 100 000 population. *FDR-adjusted *p* < 0.05.

Table 2 presents annual reported incidence rates of all syphilis subtypes stratified by sex, and Figure 2 visualises joinpoint regression trend curves separated by gender. Males reached a peak total syphilis reported incidence of 131.78 per 100,000 persons in 2010, whereas females recorded a higher concurrent peak of 179.48 per 100,000 persons. Two statistically significant joinpoints were identified for overall syphilis trends in both sexes. For males, the reported incidence rose markedly from 2005 to 2007 (APC = 39.63%, 95% CI: 11.62–62.77, *p* < 0.001), followed by a moderate yet significant decline over 2007–2021 (APC = −3.17%, 95% CI: −5.25 to −1.59, *p* = 0.003), and an apparent sharp reduction during 2021–2025 (APC = −28.77%, 95% CI: −38.65 to −21.67, *p* < 0.001). The full-period AAPC for male total syphilis was −5.54% (*p* < 0.001). Among females, the reported incidence increased significantly from 2005 to 2009 (APC = 18.59%, 95% CI: 7.75–44.90, *p* < 0.001), declined moderately between 2009 and 2019 (APC = −7.19%, 95% CI: −10.97 to −4.03, *p* < 0.001), and showed an apparent steeply from 2019 to 2025 (APC = −29.47%, 95% CI: −35.37 to −25.39, *p* < 0.001). The female AAPC stood at −10.23% (*p* < 0.001), indicating a faster long-term population-level decline relative to males.

**Table 2:** Reported incidence of syphilis stratified by Sex in Xihu district, Hangzhou, China, 2005–2025.

Year	Male-IR						Female-IR					
	All syphilis	Primary syphilis	Secondary syphilis	Tertiary syphilis	Latent syphilis	Congenital syphilis	All syphilis	Primary syphilis	Secondary syphilis	Tertiary syphilis	Latent syphilis	Congenital syphilis
2005	63.06	16.09	13.51	0.00	33.14	39.14	74.62	9.07	19.83	0.00	45.71	0.00
2006	81.75	19.22	23.13	0.33	37.46	188.11	106.14	12.43	23.85	0.00	68.18	180.25
2007	112.61	34.88	29.23	0.00	47.50	112.36	119.56	18.61	25.15	0.00	74.08	181.23
2008	122.97	53.12	24.27	0.00	45.25	37.13	135.60	31.95	24.47	0.00	78.84	35.92
2009	130.71	50.61	31.17	0.00	48.60	39.09	143.02	36.11	24.54	0.00	81.68	76.22
2010	131.78	54.38	24.02	1.33	51.71	40.73	179.48	45.82	30.90	0.00	102.41	39.34
2011	99.49	47.2	15.82	0.77	35.72	0.00	129.17	52.04	15.37	0.00	61.75	0.00
2012	78.13	32.68	15.06	0.00	30.38	0.00	128.24	37.72	22.63	0.00	67.89	0.00
2013	81.46	20.49	14.93	0.76	45.28	0.00	128.42	32.17	17.69	0.00	78.56	0.00
2014	96.91	14.81	21.34	0.75	60.00	0.00	106.58	10.39	18.38	0.00	77.53	37.94
2015	99.01	6.90	22.66	1.48	67.98	0.00	96.47	5.69	13.82	0.27	76.42	38.58
2016	93.82	6.17	14.07	1.98	71.60	0.00	92.42	2.07	10.35	0.26	79.47	28.72
2017	96.74	3.88	12.85	0.73	79.28	0.00	87.86	3.81	9.40	0.25	74.40	0.00
2018	82.02	5.10	10.19	1.85	64.88	0.00	80.69	2.19	7.29	0.24	70.97	0.00
2019	86.36	2.60	8.90	1.74	73.13	0.00	82.28	2.73	5.20	0.50	73.86	0.00
2020	75.07	3.36	7.57	0.84	63.29	0.00	59.49	1.15	1.15	0.23	56.96	0.00
2021	68.11	3.57	9.63	0.71	54.20	0.00	35.20	0.95	3.60	0.00	30.66	0.00
2022	51.95	4.86	7.82	0.87	38.40	0.00	21.46	0.38	3.95	0.00	17.13	0.00
2023	50.90	3.28	8.11	0.52	39.00	0.00	26.55	0.37	3.55	0.56	22.06	0.00
2024	28.79	2.56	4.09	0.17	21.97	0.00	12.00	0.37	1.66	0.18	9.78	0.00
2025	16.83	0.99	1.82	0.33	13.70	0.00	9.29	0.00	0.88	0.18	8.24	0.00

IR, reported incidence rate. “All syphilis” comprises primary, secondary, tertiary, and latent syphilis. Reported incidence rates for non-congenital syphilis subtypes are calculated per 100,000 population. The standalone congenital syphilis notification rate is calculated per 100,000 live births.

Notable sex-based disparities in reported incidence trends were observed across all clinical syphilis stages. Primary syphilis presented striking gender gaps in long-term epidemic trajectories. Three joinpoints partitioned the temporal trajectory of male primary syphilis: a significant rising phase from 2005 to 2010 (APC = 32.46%, 95% CI: 15.45–56.21, *p* < 0.001), a steep significant decline spanning 2010–2017 (APC = −34.11%, 95% CI: −46.34 to −27.35, *p* < 0.001), a flat non-significant period from 2017 to 2023, and a dramatic significant reduction in 2023–2025 (APC = −46.01%, 95% CI: −62.04 to −22.28, *p* < 0.001), with an overall AAPC of −12.80% (*p* < 0.001). By contrast, female primary syphilis had two joinpoints: non-significant mild growth from 2005 to 2010 (APC = 42.16%, 95% CI: −2.70 to 279.17, *p* = 0.076), sustained significant decline across 2010–2023 (APC = −30.88%, 95% CI: −42.33 to −21.16, *p* < 0.001), and an extreme sharp drop during 2023–2025 (APC = −91.55%, 95% CI: −95.62 to −68.32, *p* < 0.001). Its full-period AAPC reached −32.91% (*p* < 0.001), far outpacing the downward rate seen in males.

Gender divergence was also prominent in secondary syphilis trends. Male secondary syphilis exhibited two joinpoints: rapid significant growth from 2005 to 2007 (APC = 43.60%, 95% CI: 3.36–84.53, *p* = 0.012), a gradual significant decline over 2007–2023 (APC = −8.29%, 95% CI: −10.60 to −6.31, *p* < 0.001), and a final sharp significant decrease from 2023 to 2025 (APC = −49.30%, 95% CI: −60.55 to −28.92, *p* < 0.001), with an AAPC of −9.61% (*p* < 0.001). One joinpoint at 2013 defined the trend of female secondary syphilis: the reported incidence remained stable with non-significant fluctuation from 2005 to 2013, followed by a long-term significant decline from 2013 to 2025 (APC = −20.92%, 95% CI: −54.97 to −15.89, *p* < 0.001), corresponding to an AAPC of −13.78% (*p* < 0.001).

Tertiary syphilis showed wide annual fluctuations in both sexes with narrow absolute case numbers. For males, the 21-year unified APC was 31.91% (95% CI: 2.96–67.85, *p* = 0.027); for females, the unified APC was marginally higher at 35.58% (95% CI: 11.20–64.97, *p* = 0.008). Both estimates rely on minimal case counts and broad confidence intervals, so they only reflect percentage-level numerical variation rather than confirmed steady growth of late syphilis infections in the population. Latent syphilis showed distinct timing and magnitude of decline between men and women. Male latent syphilis contained a single joinpoint in 2020: slow significant growth occurred from 2005 to 2020 (APC = 5.12%, 95% CI: 2.57–8.15, *p* = 0.006), followed by a steep significant decline between 2020 and 2025 (APC = −26.62%, 95% CI: −38.27 to −17.93, *p* < 0.001), with an overall AAPC of −3.91% (*p* = 0.011). Female latent syphilis had one joinpoint at 2019: the reported incidence stayed steady without significant change from 2005 to 2019, then declined rapidly and significantly from 2019 to 2025 (APC = −32.22%, 95% CI: −39.15 to −26.28, *p* < 0.001), yielding an AAPC of −10.48% (*p* < 0.001).

### Age-stratified reported incidence and temporal trends of syphilis

**Figure 3:**
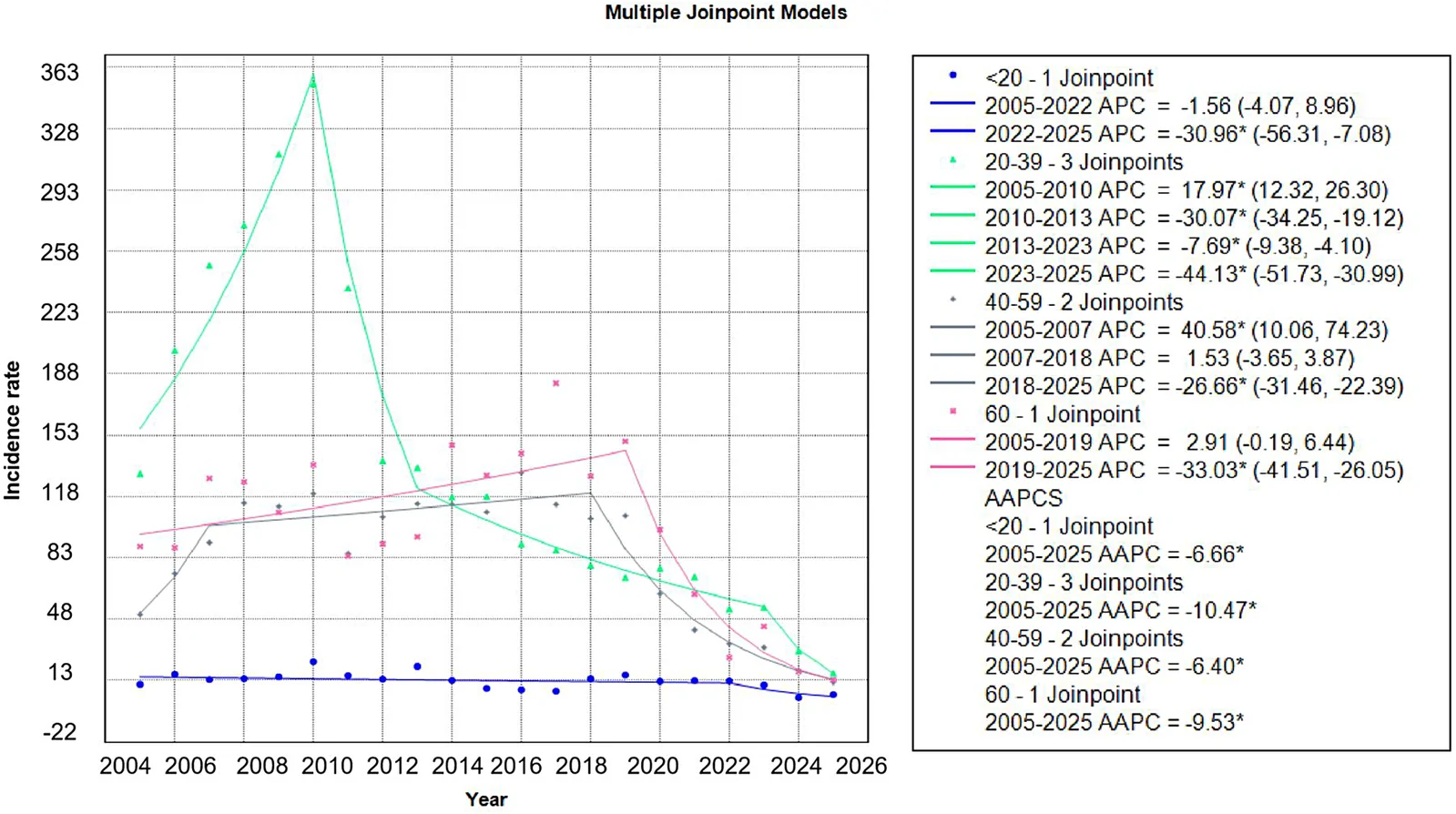
Age-specific epidemiological trends of reported syphilis incidence in xihu district, Hangzhou, China, 2005–2025. * FDR-adjusted *p* < 0.05.

Annual syphilis incidence rates were analysed across four mutually exclusive age subgroups: <20 years, 20–39 years, 40–59 years, and ≥60 years. Table 3 summarises age-stratified the reported incidence data, while Figure 3 visualises joinpoint regression trends for each age cohort. Marked inter-group heterogeneity in disease burden and epidemic timing was detected. The 20–39 age group sustained the heaviest syphilis burden throughout the surveillance period; its reported incidence climbed to a peak of 353.39 per 100,000 persons in 2010 and then fell steadily to 17.39 per 100,000 by 2025. The elderly cohort aged ≥60 years exhibited a delayed epidemic peak of 182.66 per 100,000 in 2017. In contrast, adolescents under 20 years consistently recorded the lowest reported incidence levels over the 21-year study window. Each age bracket displayed unique segmented temporal trends identified via joinpoint modelling.

**Table 3:** Reported incidence of syphilis stratified by Age group (per 100,000 population) in Xihu district, Hangzhou, China, 2005–2025.

Year	<20-IR (per 100,000 population)	20–39-IR (per 100,000 population)	40–59-IR (per 100,000 population)	≥60-IR (per 100,000 population)
2005	10.85	131.27	50.78	89.62
2006	16.68	201.56	74.05	88.93
2007	13.55	250.06	91.72	128.42
2008	14.07	272.97	114.32	126.41
2009	15.19	313.52	112.41	109.01
2010	23.76	353.39	119.78	136.06
2011	15.88	237.00	85.50	83.98
2012	13.79	138.42	106.37	91.01
2013	21.09	134.63	113.81	95.00
2014	13.00	117.85	113.54	147.36
2015	8.65	118.2	109.20	130.28
2016	7.84	90.95	131.75	142.66
2017	7.02	87.46	113.45	182.66
2018	14.11	78.83	105.39	129.74
2019	16.21	71.87	107.06	149.6
2020	12.60	77.14	62.55	99.20
2021	12.92	72.27	41.94	62.43
2022	12.71	53.85	33.98	26.24
2023	10.43	54.84	31.95	43.86
2024	3.25	30.08	19.04	18.20
2025	5.01	17.39	12.02	13.51

IR, reported incidence rate.

Adolescents aged under 20 years maintained low overall disease burden with a late sharp decline. One statistically meaningful joinpoint at 2022 split the trend for the <20-year group. The reported incidence remained stable with non-significant fluctuations from 2005 to 2022, followed by a pronounced significant reduction from 2022 to 2025 (APC = −30.96%, 95% CI: −56.31 to −7.08, *p* = 0.009). The full-period AAPC for this subgroup was −6.66% (*p* = 0.007).

Adults aged 20–39 years constituted the dominant high-risk population with four distinct trend phases. Three joinpoints divided the temporal trajectory of the 20–39-year population into four segments. The reported incidence rose significantly between 2005 and 2010 (APC = 17.97%, 95% CI: 12.32 to 26.30, *p* < 0.001), followed by a rapid significant decline during 2010–2013 (APC = −30.07%, 95% CI: −34.25 to −19.12, *p* < 0.001). Moderate yet significant reductions continued from 2013 to 2023 (APC = −7.69%, 95% CI: −9.38 to −4.10, *p* < 0.001), and an apparent steep annual decrease was observed for 2023–2025 (APC = −44.13%, 95% CI: −51.73 to −30.99, *p* < 0.001); the two-year segment restricts reliable trend interpretation. The full-period AAPC reached −10.47% (*p* < 0.001), representing the most substantial long-term decline among all age groups.

Middle-aged adults aged 40–59 years experienced early growth followed by a prolonged flat phase and subsequent decline. Two joinpoints characterised the trend of the 40–59-year middle-aged subgroup. The reported incidence increased rapidly and significantly from 2005 to 2007 (APC = 40.58%, 95% CI: 10.06–74.23, *p* < 0.001), then plateaued with non-significant annual changes across 2007–2018. A sustained significant downward trend emerged from 2018 to 2025 (APC = −26.66%, 95% CI: −31.46 to −22.39, *p* < 0.001), with an overall AAPC of −6.40% (*p* < 0.001).

Elderly adults aged ≥60 years showed a slow pre-2019 rising trend followed by accelerated reported incidence reduction. A single joinpoint at 2019 separated the epidemic curve for the ≥60-year cohort. The reported incidence edged upwards mildly without statistical significance from 2005 to 2019, before entering an apparent accelerated declining phase from 2019 to 2025 (APC = −33.03%, 95% CI: −41.51 to −26.05, *p* < 0.001). The full-period AAPC was −9.53% (*p* < 0.001).

### Sensitivity analyses

As the central-urban district of Hangzhou, Xihu District underwent substantial population growth over the 21-year study period and hosts a large floating-migrant population. Despite consistent matching rules between case records and population metrics, bias related to mobile migrant populations may distort estimates of reported incidence. Two opposing sources of bias exist: short-term migrants residing for fewer than six months are excluded from the resident-population denominator yet face elevated STI exposure risks, which may overestimate reported incidence; conversely, infected migrants seeking healthcare outside Xihu District are missed by local surveillance, leading to under-ascertainment of case counts. Such biases may exert greater influence during periods of high inter-district population mobility, namely 2005–2010 and post-2019.

We performed two sets of sensitivity analyses to evaluate result robustness. First, we recalculated reported incidence rates using registered household-registration (hukou) population as an alternative denominator (Supplementary Figure S1). Regardless of which population baseline was applied, the overall inverted-U epidemic trajectory and key inflection years remained consistent, with only small discrepancies in absolute reported-incidence values. These findings support the stability of our core trend conclusions. Second, we assessed joinpoint-model robustness by varying the maximum permitted number of joinpoints (range: 1–2) (Supplementary Figure S2). Major inflection-point years and segmented trend directions were preserved under different model constraints, confirming the reliability of our final model outputs.

## Discussion

This study provides a comprehensive 21-year epidemiological analysis of syphilis in Xihu District, Hangzhou, revealing a remarkable trajectory from a rapidly escalating epidemic in the 2005 toward sustained decline in recent years. The findings highlight several key epidemiological features, including the dominant role of latent syphilis in sustaining notified cases, the absence of notified congenital syphilis cases after 2017, the shifting gender dynamics with emerging male predominance, and the persistent burden among young adults and elderly populations. These results carry important implications for syphilis control strategies.

The substantial reduction in syphilis reported incidence observed in Xihu District since 2010 contrasts with national-level trends that showed continued increases through 2020 (4, 14). An age-period-cohort analysis covering eastern China from 2005 to 2024 further verifies that the reported incidence of infectious syphilis has declined across cities in Zhejiang Province (15). This local success stands in even starker contrast to the global epidemiological landscape: a comprehensive 2025 systematic review estimated approximately 8 million new syphilis cases among adults aged 15–49 years worldwide in 2022, accompanied by roughly 700,000 congenital syphilis cases, with sustained upward trajectories documented across North America, Europe, and multiple Asian regions (16). A study on the spatiotemporal heterogeneity of syphilis transmission across China noted that eastern cities with sound healthcare and screening systems tend to hit epidemic inflection points earlier, whereas central-western regions experience longer incidence rises due to insufficient service coverage and gaps in migrant-population management (14). This pattern provides one plausible hypothetical explanation for the diverging syphilis trajectories observed between Xihu District and the global context. As an economically developed locality in eastern China, Xihu District may have benefited from adequate medical resources and favourable policy implementation [such as China's “National Action Plan for Syphilis Prevention and Control (2010–2020)” rolled out in the early 2010s] (17), which could have contributed to falling incidence rates, though the present study did not empirically evaluate these factors.

The substantial decline in notified syphilis after 2019 requires cautious interpretation due to statistical limitations and pandemic interference. The 2023–2025 trend segment only includes two annual observations; this segment lacks residual degrees of freedom, so its APC of −39.01% cannot be treated as a reliable long-term trend. The 2019–2023 segment also has a wide 95% CI (−23.26 to −6.68), indicating low estimation precision. Domestic national surveillance evidence further confirms the reporting bias of this downward trend: Wu et al. (2022) (18) found China's syphilis notifications dropped 13.32% in 2020 and 10.41% in 2021, 18%–20% lower than predicted levels, and warned such falls did not equal real infection reduction. COVID-19 mobility curbs and suspended routine screening suppressed case identification, while asymptomatic high-risk groups avoided medical consultation (19). The observed downturn in Xihu District likely reflects, at least in part, similar surveillance artifacts.

The decline in primary and secondary syphilis rates is particularly encouraging, as these stages reflect recent infections and are most amenable to prevention efforts. The 99% reduction in primary syphilis from its peak suggests improved case detection and timely treatment. Joinpoint regression modelling identified no statistically significant trend inflection point for tertiary syphilis, which maintained a persistent upward AAPC of 31.37% over the entire observation window. This numerically positive APC value may hypothetically relate to long-term cumulative impacts of incompletely followed-up early syphilis cases, in which delayed or insufficient antibiotic treatment could theoretically lead to progression to cardiovascular or neurosyphilis (20). However, this inference is weakened by the extremely small absolute volume of tertiary syphilis cases and unstable trend estimation; we cannot confirm this causal link solely based on current surveillance data. The trend of tertiary syphilis observed in the present study differs from the provincial-wide analysis by Wu et al. (2023) (7). Two factors may partly explain this inconsistency. First, this research only relies on surveillance data from Xihu District, where the annual number of tertiary syphilis cases is extremely low. Isolated sporadic cases in individual years can drastically inflate percentage-based trend estimates, leading to prominent fluctuations driven by small sample bias. Second, the provincial study aggregates data across numerous counties and districts with a far larger total case pool, which offsets minor annual numerical variations and yields a declining trend for tertiary syphilis. The increasing proportion of latent cases—while reflecting the natural predominance of asymptomatic infections—also indicates enhanced screening activities that detect infections that might otherwise remain undiagnosed. A global epidemiological shift from symptomatic primary/secondary syphilis to asymptomatic latent infection has also been validated by cross-national surveillance data (21), indicating that symptom-based passive case reporting cannot fully capture transmission risks.

The reported incidence of congenital syphilis shows marked year-to-year fluctuations and should be interpreted with caution. The extreme peak observed in 2006–2007 may partly reflect overreporting and misdiagnosis: Wu et al. (NEJM, 2010) (22) reported that in a 2008 survey conducted in Shanghai, all 42 infants reported as having congenital syphilis (CS) tested negative for both Rapid Plasma Reagin Test (RPR) and Treponema Pallidum Particle Agglutination test (TPPA) serological tests after 3–10 months of follow-up, indicating a large number of false-positive reports under relatively lenient diagnostic criteria. The temporary zero of notified cases between 2011 and 2013 coincided with the nationwide scale-up of standardized antenatal syphilis screening, standardized diagnostic protocols, and strengthened case verification procedures, which effectively reduced false-positive reports and improved maternal treatment coverage. China issued the China 2010–2020 Plan for Syphilis Control and Prevention in June 2010 (17), which stipulated a core goal of bringing down congenital syphilis incidence below 15 per 100,000 live births by 2020 via dual coordinated control of Mother-to-child transmission (MTCT) of syphilis and HIV. Nevertheless, moderate resurgence of notified cases was observed from 2014 to 2016. This rebound may be epidemiologically explained by the growing population of migrant pregnant women: a proportion of these women obtained prenatal care outside Xihu District and received incomplete screening or delayed treatment before delivery locally. Fragmented cross-regional maternal healthcare weakened timely intervention, reintroducing risks of vertical transmission and contributing to newly detected congenital syphilis cases.

Surveillance data show that no cases of congenital syphilis have been reported in Xihu District since 2017; however, the absence of reported cases cannot be taken as evidence that mother-to-child transmission has been completely eliminated. The updated national Action Plan for the Elimination of Mother-to-Child Transmission of AIDS, Syphilis and Hepatitis B (2022–2025) further expanded antenatal screening and partner testing requirements (23), providing continuous policy support to sustain the zero-case status in urban Zhejiang. Studies have shown that the proportion of pregnant women initiating syphilis treatment within 14 weeks of gestation increased from 12.4% in 2013 to 50.81% in 2019 (24). Early diagnosis and treatment during the first trimester can reduce adverse pregnancy outcomes associated with maternal syphilis, providing clinical evidence to support current measures to prevent mother-to-child transmission (24).

The observed shift from female predominance in early study years to male predominance from 2021 onward is noteworthy. This pattern mirrors national STI surveillance data, which showed that syphilis incidence was higher in females than males between 2006 and 2020 before being overtaken by males in 2021–2022 (4). The increasing male predominance may reflect higher risk behaviors among men, including unprotected sex with commercial sex workers and multiple sexual partners (25). A case-control study focusing on eastern Chinese who have sex with men (MSM) populations identified high rates of polysexual partnerships, recreational drug co-use, and avoidance of STI screening due to stigma as key drivers of persistent syphilis transmission among men (26). Additionally, the availability of drugs for erectile dysfunction may enable older men to remain sexually active, contributing to the rising male burden in elderly populations (27, 28). The Global Burden of Disease 2019 analysis confirmed that extended sexual activity among older males significantly elevated STI infection risks across high-income Asian-Pacific regions (29). As these factors are not directly measurable in routine surveillance, they warrant targeted behavioural research. Stratified analysis by clinical subtype further amplified gendered differences: female primary syphilis exhibited a far more pronounced long-term decline (AAPC = −32.91%) compared with males (AAPC = −12.80%), likely reflecting greater healthcare access and screening exposure among women (30, 31). In contrast, males typically only accessed clinical services following the emergence of overt genital lesions, leading to prolonged infectious windows before diagnosis (32). Calculated AAPC values for tertiary syphilis were positive for both sexes, with a slightly higher figure in females (35.58%) than males (31.91%). This numerical gap may potentially suggest residual historical untreated latent infections among women, but the tiny annual case counts and wide confidence intervals make this speculation tentative and unvalidated (33). Latent syphilis also declined faster in females (AAPC = −10.48%) than males (AAPC = −3.91%), a pattern documented elsewhere in eastern China (15). Future efforts should expand accessible syphilis screening for underserved middle-aged, elderly male and MSM groups (34).

Joinpoint regression outputs across four predefined age strata demonstrated heterogeneous epidemic trajectories, with the 20–39 age cohort constituting the dominant high-risk population sustaining the highest reported incidence levels throughout the 21-year observation period, peaking at 353.39 per 100,000 inhabitants in 2010. This finding is consistent with domestic and international studies on sexually transmitted infections. According to the global burden analysis of sexually transmitted infections from 1990 to 2019, the peak reported incidence of syphilis was concentrated within the 20–39 years sexually active population (35); the global high-risk age segment gradually shifted from 25 to 29 years in 2010 to 20–24 years in 2019, while the 30–34 years group contributed the largest volume of new infections (36, 37). This population aged 20–39 comprises college students, young floating populations, and service sector workers, who commonly exhibit characteristics such as frequent changes of sexual partners, suboptimal condom use, and insufficient access to formal sexuality education in schools (38). In addition, the increasing ubiquity of social media and mobile dating applications may further heighten their vulnerability to high-risk sexual contacts (38). Comprehensive intervention packages for this high-burden population require cross-sector collaboration between tertiary education institutions, youth community centres, and local CDCs (39).

The geriatric population aged ≥60 years represented another vulnerable subgroup with prolonged rising reported incidence (APC = 2.91%) during 2005–2019 in this study. This fact is further corroborated by a study conducted in China, 48.8% of reported syphilis cases in 2019 occurred in individuals over 50 years of age (40). Parallel upward trends in geriatric syphilis detection rates have been well-documented (41, 42) across North America, Europe, South Korea, and Brazil, where a national ecological time-series study reported an annual 25.0% growth in elderly syphilis notifications between 2011 and 2019 (APC = 25.0, 95%CI: 22.1–28.1) (27). Multiple interrelated social and physiological factors may contribute to this global trend: extended life expectancy preserving sexual demand among older individuals, pervasive societal misconceptions regarding asexuality in ageing populations, extremely low routine condom uptake, thinning vaginal mucosa and reduced immune function and historical exclusion of syphilis serology from standard geriatric physical examinations and chronic disease management (27, 43–45) This underscores the need for routine syphilis screening in geriatric populations, which is currently not standard practice in most Chinese healthcare settings. This study bears distinct methodological strengths alongside acknowledged limitations. Primary strengths include an uninterrupted 21-year population surveillance dataset capturing the complete epidemic cycle of syphilis from emergence to sustained suppression, multi-dimensional stratification by clinical stage, gender and age to disaggregate heterogeneous subgroup trends, standardised joinpoint regression modelling to quantitatively segment temporal shifts and identify statistically significant epidemic inflection points, filling a regional research gap in long-term syphilis epidemiological surveillance. Key limitations stem from the passive notifiable disease surveillance design, which inherently underestimates true population-level syphilis prevalence due to unreported asymptomatic latent infections, underascertainment at grassroots private clinics, and care avoidance driven by STI-related social stigma; this reporting bias is ubiquitous within national passive surveillance systems across low- and middle-income countries. Second, aggregated annual reported incidence data lack individual-level behavioural covariates including condom adherence, number of sexual partners, and cross-regional migration duration, prohibiting multivariate risk factor regression to establish causal inference between social determinants and syphilis reported incidence fluctuations. Third, the single-district ecological design restricts cross-jurisdictional comparative analysis and cannot quantify the contribution of intercity population mobility to local syphilis importation and transmission. Future multi-centre cohort research combining individual behavioural questionnaires and cross-district surveillance linkage is recommended to unpack modifiable social and behavioural risk factors shaping regional syphilis dynamics.

Four major temporal inconsistencies in national surveillance frameworks introduced systematic bias to long-term trend estimation. First, the national syphilis diagnostic standard was updated from GB 15974-1995, WS 273-2007 to WS 273-2018, which may have altered case definitions and staging criteria. Second, the rollout of the National Syphilis Prevention and Control Plan (2010–2020) expanded universal screening across inpatient, preoperative, antenatal and blood donor groups, artificially boosting identification of asymptomatic latent syphilis and elevating reported incidence without a corresponding change in true incidence. Third, the nationwide internet-based direct reporting system for infectious diseases was still being phased in and refined after 2003, so reporting coverage and data quality in the early years (e.g., 2005) were likely lower than in later years. Fourth, published domestic studies have validated that nationwide surges in reported syphilis largely stemmed from changes in diagnostic and reporting practices rather than true epidemic growth. Consistent with Liu et al. (46) and Wu et al. (22), the 25.23% APC recorded for 2005–2010 may partly reflect surveillance-system maturation and screening expansion, instead of a genuine rise in local syphilis prevalence.

## Conclusion

Collectively, despite the prominent long-term descending trend of overall syphilis reported incidence and the absence of notified congenital syphilis cases in Xihu District after 2017, three major public health challenges remain unaddressed: heavy transmission burden among young adults, insufficient screening coverage for middle-aged and elderly males, and a large hidden pool of undiagnosed latent syphilis carriers sustaining ongoing transmission. Sustained multi-pronged interventions are required, encompassing differentiated population-targeted screening protocols, full-cycle serological follow-up for latent syphilis patients, permanent universal antenatal MTCT prevention, embedded syphilis testing within routine primary care physical examinations, and seasonal pre-summer intensified STI outreach campaigns aligned with the documented March–August national high-incidence window of syphilis across Zhejiang Province (4). Continuous annual joinpoint trend monitoring should be maintained to dynamically adjust targeted prevention strategies in response to emerging epidemic inflection points.

## Data Availability

The raw data supporting the conclusions of this article will be made available by the authors, without undue reservation.
