# Supplementary material for: Epidemiological trends of reported syphilis incidence in Xihu district, Hangzhou, China, 2005–2025
Source: Front Epidemiol. 2026 Sep 11;6:1944790. doi: 10.3389/fepid.2026.1944790 (PMC13613378; doi:10.3389/fepid.2026.1944790)
Supplement: Supplementary file 1 [file Datasheet1.docx]

**SUPPLEMENTAL MATERIAL**

**Epidemiological Trends of Reported** **Syphilis Incidence in Xihu District, Hangzhou, China, 2005–2025**

**Appendix:**

**Figure S1.** Reported incidence rates of syphilis calculated using registered household‑registration (hukou) population as the alternative denominator, 2005‑2025.

**Figure S2.** Joinpoint regression outputs varying the maximum permitted number of joinpoints (range: 1‑2), 2005–2025.

Figure S1. Reported incidence rates of syphilis calculated using registered household‑registration (hukou) population as the alternative denominator, 2005‑2025. * FDR-adjusted *p* < 0.05.

Figure S2. Joinpoint regression outputs varying the maximum permitted number of joinpoints (range: 1‑2), 2005–2025. Panel A: maximum of 1 joinpoint; Panel B: maximum of 2 joinpoints. * FDR-adjusted *p*< 0.05.
